# Novel SMARCA4 Variant in an Infant With Atypical Teratoid Rhabdoid Tumor

**DOI:** 10.1097/MPH.0000000000003004

**Published:** 2025-03-03

**Authors:** Shria K. Haldipurkar, Sudarshawn N. Damodharan, Pamela Rathbun, Kai Lee Yap, Nitin Wadhwani, Angela J. Waanders

**Affiliations:** * Division of Hematology, Oncology, Neuro-Oncology and Stem Cell Transplant, Ann & Robert H. Lurie Children’s Hospital of Chicago; ‡ Department of Pathology and Laboratory Medicine, Ann & Robert H. Lurie Children’s Hospital of Chicago; † Department of Pediatrics, Section of Hematology, Oncology, and Stem Cell Transplant, University of Chicago, Comer Children’s Hospital, Chicago, IL

**Keywords:** ATRT, molecular, infant, CNS, variant, genetic

## Abstract

Atypical teratoid/rhabdoid tumors (AT/RT) are malignant central nervous system (CNS) tumors. Typically, AT/RT is classified as SMARCB1 (INI-1) deficient or as SMARCA4 (BRG1) deficient. In this case, we describe a unique case of AT/RT with a novel SMARCA4 missense variant identified on next-generation sequencing but retained expression of INI-1 and BRG-1 on immunohistochemistry. Diagnosis of the tumor and discovery of the novel SMARCA4 variant was only possible after comprehensive tumor molecular testing tailored for pediatric malignancies. This case highlights the importance of molecular genetic testing as part of a workup in neoplasms concerning for possible AT/RT.

Atypical rhabdoid/teratoid tumors (AT/RT) are malignant embryonal tumors of the central nervous system (CNS). AT/RT constitutes <2% of all pediatric CNS tumors and is most commonly present in early childhood. Such diagnoses account for 40% to 50% of all CNS malignancies within the first year of life, with the median age of diagnosis being 16 to 30 months.^1^ These tumors are aggressive with disseminated disease present at diagnosis in 20% to 30% of cases.^1^ Given this, treatment is typically multimodal, with a combination of surgery, chemotherapy, and radiation.

Historically, AT/RTs were misclassified as primitive neuroectodermal tumors (PNETs) due to their indistinguishable radiographic and histologic similarities. It was introduced as its own entity within the World Health Organization (WHO) classification of CNS tumors in 2000.^2^ Currently, AT/RT is typically distinguished by immunohistochemical (IHC) staining demonstrating a loss of INI1 expression.^3^ We now know that most AT/RTs harbor pathogenic variants in the SMARCB1 (INI-1) gene at chromosomal band 22q11.2.

Loss of SMARCB1 disrupts activation of the SWI/SNF chromatin remodeling complex, which plays a crucial role in cell differentiation, leading to dysregulation of gene expression and tumor suppression.^4^ AT/RTs, harboring SMARCB1 alterations, can be subdivided into 3 molecular groups based on DNA methylation profiles: AT/RT-TYR, AT/RT-SHH, and AT/RT-MYC. These subgroups differ in gene expression and activation of signaling pathways. This has impacted our understanding of these tumors’ biology, with potential avenues opened for therapeutic targeting based on the genetic information of the tumor.

In rare cases, SMARCA4 (BRG-1) variants have also been described in AT/RT. Individuals with AT/RT-SMARCA4 are younger in age, more likely to be carriers of germline mutations and have shorter overall survival.^4^ Moreover, DNA methylation studies show that AT/RT*-*SMARCA4 does not classify in any of the known AT/RT subgroups and should be regarded as a distinct entity.^4^ There is inadequate knowledge regarding the specific mechanisms involved in AT/RT-SMARCA4 development, making it increasingly difficult to treat.

Here, we present a case of AT/RT retaining expression of both INI-1 and BRG-1 on immunohistochemistry, which led to a difficult diagnosis, along with findings of a novel SMARCA4 missense variant. In this report, we demonstrate the importance of molecular diagnostic testing and germline testing as a follow-up to somatic profiling that can impact clinical care.

## RESULTS

### Case Presentation

**FIGURE 1:**
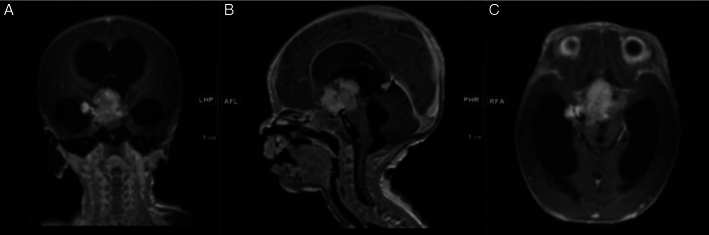
Selected MRI images. T2-weighted MRI head imaging was obtained at the time of our patient’s initial presentation. A, Coronal viewpoint of our patient’s 2.0 ×1.9 ×2.2 cm lesion located along the right suprasellar/interpeduncular cistern. B, Sagittal viewpoint. C, Axial viewpoint with mass effect on the right prechiasmatic optic nerve.

**FIGURE 2:**
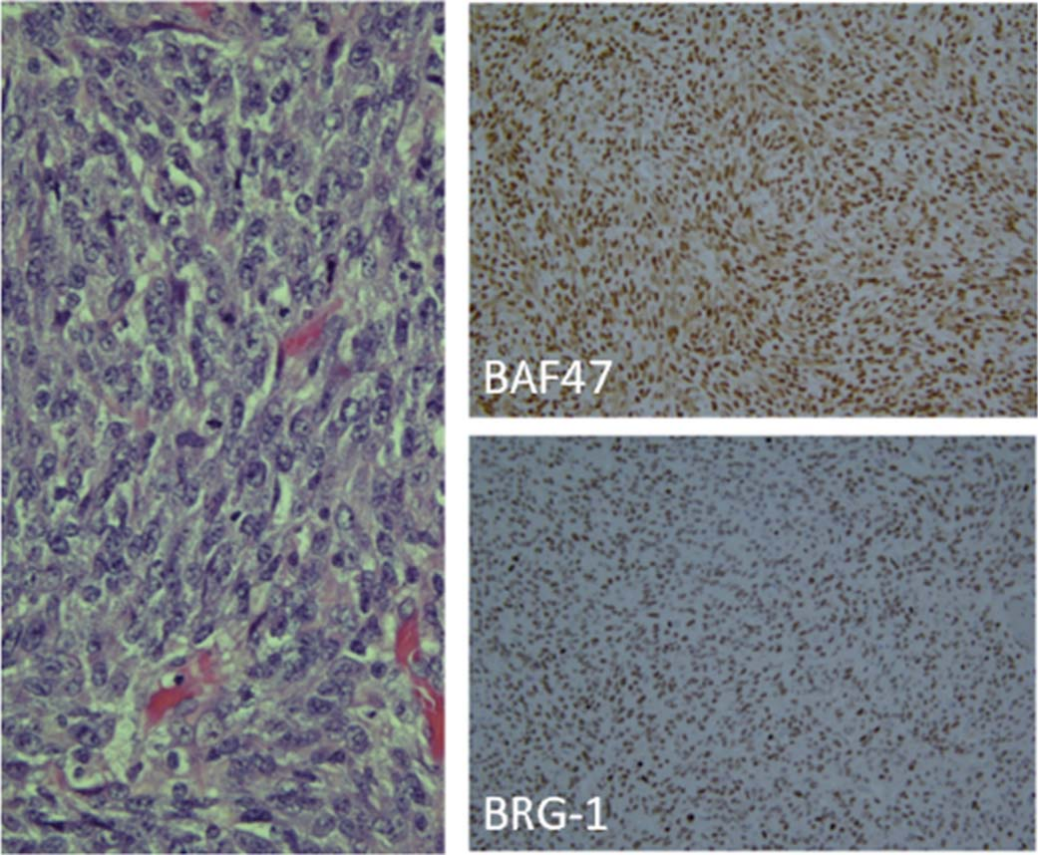
Histopathology slides. H&E-stained slides demonstrate a epithelioid to spindled cell neoplasm with increased mitotic activity and intracytoplasmic eosinophilic material, biopsy of the suprasellar brain tumor histologically categorized as a primitive spindle neoplasm. Immunostains were performed and showed the tumor cells to be focally positive for EMA, S-100, GFAP, SALL4, synaptophysin, Olig2, and CD99. INI-1 (BAF47) and BRG-1 are retained as depicted above.

A previously healthy 2-month-old girl, born at 39 weeks of gestation, was admitted to the hospital for failure to gain weight. The initial physical examination was significant for bilateral eye swelling and CNIII palsy. As part of her initial evaluation, a screening head ultrasound was done and an intracranial mass was identified. Magnetic resonance imaging (MRI) of the head confirmed the presence of a large, centrally located tumor (Fig. 1). The patient underwent a biopsy with initial pathology consistent with a high-grade embryonal type neoplasm based upon histology. Initial IHC done showed retention of both INI-1 and BRG-1 (Fig. 2). Tumor molecular testing was initially performed with an adult malignancy-focused somatic panel and no clinically significant variants were reported. The patient was transferred to a tertiary hospital for further care. Repeated NGS testing, using a pediatric malignancy-focused panel, at our site detected a SMARCA4 missense variant along with methylation consistent with AT/RT, WHO Grade 4 (Table 1).

**TABLE 1:** Findings of Genetic Testing

Specimen tested	Gene	Associated germline disease (inheritance)	Variant information	Variant allele frequency or Zygosity	Classification
Tumor	SMARCA4	Rhabdoid tumor predisposition syndrome 2 (autosomal dominant)	Chr19(GRCh38):g.11012977T>C NM_001387283.1:c.2303T>C p.Leu768Pro	90.2%	Tier II: variants of potential clinical significance
	TET2	Immunodeficiency (autosomal recessive)	Chr4(GRCh38):g.105275706C>G NM_001127208.3:c.5196C>G p.His1732Gln	48.3%	Tier III: variants of unknown clinical significance
Peripheral blood	SMARCA4	Rhabdoid tumor predisposition syndrome 2 (autosomal dominant)	Chr19(GRCh38):g.11012977T>C NM_001387283.1:c.2303T>C p.Leu768Pro	Heterozygous	Likely pathogenic
	SMARCB1	Rhabdoid tumor predisposition syndrome 1 (autosomal dominant)	Entire gene duplication	Heterozygous	Uncertain significance

Given the concern for an aggressive high-grade CNS neoplasm, systemic chemotherapy was initiated per Baby POG regimen with vincristine, cyclophosphamide, etoposide, and cisplatin. Repeat head imaging obtained after the first course of chemotherapy demonstrated a response with a decrease in overall tumor burden. However, the patient experienced clinical deterioration during the second course with interval MRI obtained, which demonstrated intraventricular hemorrhage and hydrocephalus with mass effect on the brainstem. An external ventricular drain (EVD) was placed and eventually converted to a ventriculoperitoneal shunt (VPS) to help with cerebrospinal fluid diversion. In addition, the patient developed panhypopituitarism and new onset seizures. Following the development of dysautonomia, the patient was transferred to the PICU for further monitoring. Our patient received in total 3 courses of systemic treatment per the Baby POG regimen.

In the PICU, the patient developed pseudomonas pneumonia and was found to have severe pulmonary hypertension. The patient rapidly deteriorated with increased work of breathing that led to respiratory failure and emergent intubation. After the onset of cardiopulmonary distress and severe hypertension, no further tumor-directed therapy was attempted, and the focus shifted to palliative management. Our patient ultimately passed away ∼4 months after her initial presentation.

## DISCUSSION

This case presents an atypical case of AT/RT with IHC retention of both INI-1 and BRG-1 immunostaining along with a novel germline SMARCA4 missense variant (c.2303T>C). Typically, rhabdoid tumors can be classified as SMARCB1 deficient by demonstrating loss of INI-1 or as SMARCA4 deficient by demonstrating loss of BRG-1. Clinical findings suggest SMARCA4-altered AT/RT are associated with a worse prognosis compared with those that are SMARCB1 deficient. In this case, the tumor retained both INI-1 and BRG-1. Initial tumor molecular testing used an adult malignancy-focused panel, which incorporates SMARCB1. The diagnosis of AT/RT and discovery of the SMARCA4 variant could only be confirmed after further molecular testing at our site using a pediatric malignancy-focused panel. This highlights the importance of comprehensive molecular testing.

Previous literature has found at least 2 patients with AT/RT who retain INI-1 and BRG-1 who have been described as carriers of a missense variant in the SMARCA4 gene.^4–6^ In both cases, the missense variant was homozygous due to copy-neutral loss of heterozygosity (LOH) on chromosome 19p, encompassing the SMARCA4 locus.^5^ In addition, these variants were in the conserved DEXH-box ATPase domain or the chromatin remodeling domain suggesting that these variants could incorporate normally into the SWI/SNF chromatin remodeling complex but could compromise the chromatin remodeling activity of SMARCA4/BRG1.^2^  Like our patient, the other cases described also succumbed to their disease with an abrupted clinical course speaking to the aggressive phenotype of this tumor with SMARCA4 alteration.

Previous studies have found that approximately one-third of children with AT/RT have germline alterations of SMARCB1 predisposing them to malignant rhabdoid tumors: rhabdoid tumor predisposition syndrome (RTPS).^5^ Cases of germline mutations in rhabdoid tumors are typically followed by a fatal course. RTPS commonly occurs when genes of the SWI/SNF complex are mutated. There is strong evidence that SMARCB1 is not the only SWI/SNF complex member. Given that SMARCA4/BRG1 plays a significant role in the chromatin remodeling complex by carrying out ATPase activity, it is also involved in RTPS.^7^  Since RTPS includes manifestations of other cancers, genetic counseling is recommended for cases of AT/RT to ensure proper family counseling.

Somatic tumor molecular testing revealed a unique alteration in SMARCA4 NM_001128844.1 Exon 17 p.Leu768Pro (c.2303T>C) which has yet to be evaluated thoroughly. Upon review, our patient’s SMARCA4 variant is documented in the single nucleotide polymorphism database (dbSNP, rs769701819) but has otherwise not been reported within the literature or other larger clinically relevant genomic databases (Genome Aggregation Database, Clinvar, COSMIC, or the St. Jude PeCan). The SMARCA4 protein (also known as BRG-1) is a catalytic subunit of the SWI/SNF complex that is involved in chromatin remodeling which is found in various malignancies, including AT/RT.

In comparison to SMARCB1*-*deficient AT/RT, SMARCA4*-*mutated AT/RT is associated with a worse prognosis and a higher frequency of inherited germline alterations.^5^ This worsens prognosis by predisposing patients to early-onset cancers, disrupting cell-cycle regulation, and fueling the tumor’s aggressive nature. Clinically, our patient’s rapid deterioration after the development of pulmonary hypertension (PH) ultimately led to the discontinuation of tumor-directed treatment. This is puzzling as the exact physiology for this occurrence is not completely known. PH typically results in an increase in systemic blood pressure and eventual heart failure.^8^ Literature suggests PH is a chronic, progressive disease that may occur from cancer burdens and therapy, though more often in the adult population.^8^  Most often it is multifactorial in etiology, thought to be contributed by the interplay of a susceptible genetic background, epigenetic changes and injurious events.^9^ Previous studies suggest that during embryonic development, functional genes are spatiotemporally controlled by DNA methylation mechanisms.^10^ Similarly, changes of DNA methylation state are known to have close relation with human diseases like cancer.^10^ While there have been some anecdotal cases that may suggest SMARCBI/SMARCA4 alterations may play a role in PH, the biology of this is poorly understood, and it is likely that for our patient the PH does not have a strong genetic etiology and rather more due to the stress the body was under given the aggressive CNS neoplasm along with development of pneumonia in the setting of myelosuppressive chemotherapy.

This case highlights a novel germline AT/RT missense variant in SMARCA4. Given that AT/RT is a rare pediatric CNS malignancy and has been misdiagnosed historically, further testing as part of tumor diagnosis is very important. There is a higher frequency of germline mutations in infants with malignant rhabdoid tumors. While ATRT-SMARCB1 is more frequently diagnosed, very few of these cases are inherited from a pathogenic mutation in SMARCB1 from a healthy parent.^11^ On the other hand, germline mutations in SMARCA4 are more common and have been shown to result in more aggressive tumor behavior, leading to worse prognosis.^4^ While both SMARCB1 and SMARCA4 contribute to the SWI/SNF complex, SMARCB1 has localized impact. SMARCA4 is critical for normal chromatin remodeling across tissues. Therefore, germline SMARCA4 mutations may contribute to a more systemic loss of function, leading to a worse survival outcome compared with germline SMARCB1 mutations.

Further molecular testing is key to uncovering novel variants as was the case for our patient with a unique somatic and germline alteration of SMARCA4. Genetic testing should be a part of any AT/RT workup, especially when immunohistochemistry does not yield abnormalities in INI1/BRG1 staining. It is crucial to better understand the role of these varying molecular findings on the biology of these tumors to better cater to treatments to aid future patients.

## METHODS

### Next Generation Sequencing (Pediatric Panel)

Targeted NGS panel testing interrogating for DNA sequence variants and RNA fusions was carried out. In brief, DNA analysis of 86 genes for hotspot mutations, 44 genes for full coding sequence, and 28 genes for focal high level copy number gains as well as RNA analysis of 91 genes for potential fusion transcripts was performed. cDNA and DNA samples were target amplified using the Ion AmpliSeq Library Kit in conjunction with the Oncomine Childhood Cancer Research Assay (ThermoFisher Scientific). Sequencing was performed on the Ion S5 sequencer and compared with the published reference sequence (Genome build GRCh37) and analyzed using Ion Reporter and a customized bioinformatics pipeline to identify clinically significant variants (SNVs, insertions/deletions, gene fusions, and copy number variants) implicated in cancer. Allele frequencies were reported as observed in the sequencing data set, without adjustment for tumor percentage. This targeted NGS panel is a clinically validated assay, in accordance with CLIA requirements. Clinically significant variants were reported according to CAP/AMP/ASCO guidelines.^12^

### Next Generation Sequencing (Adult Panel)

Cytology NGS panel testing and RNA fusions were carried out. In brief, DNA analysis for 168 genes for mutations, 145 genes for copy number variations, 3 genes for fusions, and RNA analysis of 21 genes for fusion transcripts was performed. DNA was isolated using the FFPE DNA Extraction Kit (Qiagen) and amplified using the HTP Library Preparation kit in conjunction with the Qubit fluorometric assay (ThermoFisher Scientific). Sequencing was performed on a NovaSeq. 6000 system (Illumina) and analyzed using institution-custom bioinformatics pipelines and hg19 (GRCh37) human genome reference sequence for alignment.

### Germline Targeted Testing

Genomic DNA was sequenced using massively parallel sequencing (Next generation sequencing) on the Illumina Nextseq. 550 instrument. A bidirectional sequence was obtained, analyzed, and compared with the published reference sequence (Genome build, GRCh38) of the coding regions and splice junctions. Coding and splice site regions were 100% covered at a minimum of 20x coverage. Sequence and copy number variants in the targeted genes (SMARCA4, SMARCB1) were assessed using current clinical laboratory parameters for interpretation according to ACMG guidelines (Genet Med. 2015 May;17[5]:405 24, Genet Med. 2020 Feb;22[2]:245 257). The identified SMARCA4 variant was sequenced and confirmed using bidirectional dideoxynucleotide Sanger sequencing. The SMARCB1 duplication was confirmed by array comparative genomic hybridization (aCGH) using the Agilent HD LCHv3-oligonucleotide array consisting of ∼180,000 oligonucleotides (60 mers) that represent coding and noncoding human sequences in the genome with an average probe spacing of ∼20 kb to detect gains or losses at a minimum of 500 kb or smaller for regions of clinical interest.
